# Behavioral Pattern during Dental Pain in Intellectually Disabled Children: A Comparative Study

**DOI:** 10.1155/2014/824125

**Published:** 2014-11-18

**Authors:** Muthukali Shanmugam, Vetrivel Shivakumar, Vijayarangan Anitha, Bagavathi Perumal Meenapriya, Srinivasan Aishwarya, Ramakrishnan Anitha

**Affiliations:** Department of Periodontics, Chettinad Dental College & Research Institute, IT Highway, Rajiv Gandhi Salai, Kelambakkam, Chennai, Tamil Nadu 603112, India

## Abstract

*Aim.* Children with developmental disabilities generally experience more pain than the normal children. Description of pain is generally difficult in children and more so in children with intellectual disabilities. The study aimed at evaluating dental pain in children with intellectual disabilities. *Methods.* The survey was carried out in an institution caring for intellectually disabled children to determine the oral health status and the treatment needs of the special kids. 236 children were surveyed out of which the test group is comprised of 111 intellectually disabled children and the control group had 125 normal children with age ranging between five to eighteen years. A questionnaire was presented to the caregivers to elaborate about dental pain in their wards using the dental discomfort questionnaire (DDQ+). The children were examined for dental caries and periodontal status based on the WHO indices for oral hygiene status. *Result.* Results revealed a statistically significant difference between intellectual disability and brushing, chewing, and earache. The frequency of reporting dental pain was lesser in the intellectually disabled group. *Conclusion.* Children with intellectual disability tended to report dental pain of any nature with lesser frequency than typically developing peers. They also faced greater difficulty in brushing and chewing.

## 1. Introduction

Pain is a highly complex, dynamic, subjective experience that is useful in growing children, serving to warn them of danger and limiting exposure to additional injury. Children learn to prevent and cope with pain [1]. However, untreated, acute, or recurrent or chronic pain may have significant and lifelong consequences (both physiologically and psychologically). The first step in treatment process is accurate diagnosis of the problem. Since pain is an inherently subjective phenomenon, it is often said that the gold standard for pain assessment in both children and adults is verbal reporting [1–3]. It is the only way to determine the intensity and quality of the pain present. However, reliable description in children especially in intellectually disabled (ID) children is very difficult.

Children often confuse pain with fear and anxiety due to their cognitive impairment. Children with intellectual disability have an added challenge of not being able to express the pain or verbalize it [3].

Cognitively impaired children usually rely on their caregivers to interpret their distress. Hence, to gather pain relevant information, caregivers were interviewed regarding their children for expression of pain, experience, treatment, and coping behavior. Research shows that children with developmental disabilities generally experience more pain than the normal children due to chronic systemic conditions associated with their disability [4–6]. Their pain is never properly expressed because of their lack of verbal communication. Many children have idiosyncratic behaviors such as moaning/grunting which may lead to over/underestimation of pain [6].

The aim of the study was to evaluate the dental pain in children with intellectual disabilities from a caregiver's perspective.

## 2. Materials and Methods

The study was initiated after obtaining clearance from the Institutional Ethics Committee, Chettinad Health City. Informed consent was obtained from the subjects prior to start of the study.

The study was carried out on a total of 236 children. Study group is comprised of 111 intellectually disabled children. They were residing at Vasantham institution for intellectually disabled children, Mogappair, Chennai, Tamil Nadu, India. The children had intellectual disability in the mild, moderate range [7]. The control group included 125 typically developing peers from Mambakkam, Chennai, India. All the children in the control group were of normal intellectual growth as determined by their academic evaluations.

### 2.1. Selection Criteria

1. Age group ranged from 5 to 18 years.
2. The study group has a history of intellectual disability as verified by the child's medical file or caregivers reporting.
3. Caregivers were able to understand spoken English/Tamil.
4. The control group included children who had normal intellectual development.

Four investigators were assigned to do intra- and extraoral examination on the children. The nature of the study was explained and the investigators assisted them in filling the questionnaire.

### 2.2. Questionnaire

To assess the change in behavior in intellectually disabled children during tooth ache, a Dental Discomfort Questionnaire (DDQ+) was developed. The questionnaire was drafted after extensive interviews with parents or caregivers of children with pain by Versloot et al. The DDQ+ has a good predictive value for the incidence of toothache [8]. The questionnaire was distributed to all the subjects participating in the study.

A lot of questions relating to the behavior of intellectually disabled children were added to the DDQ+. The questionnaire was filled during the interviews by the caregivers with the help of the investigators. The language used was Tamil/English. The questions were tabulated and the caregivers had to select the most appropriate option that explained the occurrence of pain/discomfort in children. The options were “never” (scored as zero), “occasionally” (scored as one), and “always” (scored as two). A total numeric mean for each child and a mean of the DDQ+ for the group were calculated.

Caregivers and investigators were also asked to rate the child's dental pain on a ten point scale where “zero” represented the absence of pain and “ten” the maximum pain imaginable [9]. The scale was converted to an ordinal scale with never, mild, moderate, and severe categories. Never represented the absence of pain (score 0). Mild pain was when the rating was between 1 and 3, moderate was between 4 and 7, and severe pain was between 8 and 10. The dental examinations were conducted using a dental explorer to detect dental caries. The caries examination was based on the “WHO” criteria according to which the DMFT/dft (decayed, missing, and filled teeth) indices were scored. A frank cavitation which is a break of 0.5 mm or more into enamel was considered as caries. Any incipient or questionable caries were not included. Any teeth filled with any restoration except sealants were considered as “filled” teeth. The “m” was not included in dft because children have mixed dentition.

### 2.3. Statistical Analysis

The statistical analysis was done using Windows SPSS software version 16. All the data was reported as mean ± standard deviation (SD). Student's *t*-test, degree of freedom, mean and standard deviation, and chi-square test were analyzed. The level of significance (*P*) was set at 0.05.

## 3. Results

### 3.1. Demographic Data

The sample investigated in this study was a total of 236 children, out of which 111 were intellectually disabled children and 125 were healthy children. The study involved 151 males and 85 females. The age group of the sample ranged from 6 to 15 years.

### 3.2. Dental Findings and Pain Data

The DMFT and dft scores of the sample investigated were tabulated (Table 1). The DMFT score was higher in intellectually disabled (ID) children. The difference in the number of filled teeth between the study and control group was negligible. The dft score was increased in normal healthy children as opposed to disabled children.

The investigators put forward questions describing the specific behavioral pattern of the children experiencing dental pain. These questions were presented to the caregivers in order to find out the perception of dental pain between the control and study group and whether it was comparable. The behavioral pattern which was specifically exhibited by the children during dental pain was documented as per the caregiver's perception. Outcome of the tabulation in this study showed that ID children had problems in brushing teeth especially the upper teeth and it was statistically significant (*P* = 0.00). The other significant change during pain included biting with the molar instead of the other teeth (*P* = 0.001). Most of the ID children reached for the cheek region while eating, a behavioral change depicting vague dental pain which they were unable to express otherwise. Typically developing children reported ear pain at night (*P* = 0.014), during daytime (*P* = 0.02), and a few while eating (*P* = 0.01). In the entire study, excessive salivation in ID children during dental pain (*P* = 0.00) was one of the most important behavioral changes observed by the caregivers. Adding to this, the other significant behavioral change was the habit of placing their hand or objects inside the mouth (*P* = 0.00) (Table 2).

## 4. Discussion

According to the International Association for Study of Pain (IASP) [1], pain is defined as “an unpleasant sensory and emotional experience associated with actual or partial tissue damage.” A reliable description of such pain is generally difficult in children and even more challenging in ID children. Intellectual disability is defined as mental retardation or impairment in the areas of development or cognitive activities [10].

Intellectual disability is “a disability characterized by significant limitations both in intellectual functioning and in adaptive behavior, which covers many everyday social and practical skills. This disability originates before the age of 18” [11]. Dental problems are more common in people with special needs, sometimes not only because of the disability itself but also because of inadequate oral health care. These special children required special health care approaches as they had very poor oral hygiene. According to research and the literature, these children had physical, mental, sensory, behavioral, cognitive, emotional, and chronic medical conditions which required health care beyond the usual measures and which involved specialized knowledge, increased awareness, attention, and accommodation. Information about pain in children with cognitive impairment was always insufficient and less reliable for a dentist [11–13]. To gather any data related to pain the investigators or health care provider had to rely on the caregiver's perception depending on the level of cognitive impairment, and pain coping and expression varied in these special children [6].

Pain is a common experience and is a part of childhood. The identification and interpretation of dental pain in severe cognitive communication and motor impairments were challenging. We concluded that there is a high incidence of dental pain and treatment needs that are not catered to. In spite of parents and caregiver's educational qualifications, dental pain is rarely understood and treated; this was due to the other major chronic systemic illnesses the children undergo. Hence, dental pain in children goes unnoticed often and they are not brought to the dentist for routine checkup. Mostly caregivers identify dental pain based on the ID children change in behavior and bring it to the notice of the healthcare provider [14]. Hence, mostly ID children reach the dentist in an acute state of pain leaving very few options to save the teeth. Even the effective treatment options are compromised due to the lack of cooperation.

The data outcome of this study indicates that ID children have problem in brushing upper and lower teeth. This was because of fear and anxiety amongst intellectually disabled children in placing a foreign object into their mouth. Children should be trained patiently to overcome these problems which require a dentist's and a caregiver's cooperation. Reaching for cheek while eating and earache at night or during meals were also common behavioral changes during dental pain. The caregivers should understand these signs and bring the children to the dentist immediately because the pain expressed in such manner is mostly of acute nature. Most of the ID children had excessive salivation and the habit of placing their hand or objects inside the mouth during dental pain. These were the highly exhibited characters in intellectually disabled children. According to researches, an increase in dental caries causes more dental pain which leads to these pain related behavior. So caregivers and dentists have to work mutually to recognize specific changes in behavioral pattern [15].

Though this study was done based on research articles specializing on pediatric dentistry and cognitive impairment in children, it still had a number of limitations which include lack of professional assessment of ID children. Future studies including the comparison between different degrees of intellectual disability could further provide more specific conclusions.

## 5. Conclusion

Intellectually disabled children had more problems in brushing upper teeth and exhibited the habit of reaching for the cheek while eating. The prevalence of reporting dental pain was more in typically developing children than in intellectually disabled children.

## Figures and Tables

**Table 1:** DMFT and dft scores in children with ID and normal children.

DMFT component	Disabled mean ± SD	Normal mean ± SD	t-value	df	P value
Decayed (D)	0.64 ± 1.32	1.06 ± 1.40	2.33	234	0.02
Missing (M)	0.68 ± 2.23	0.03 ± 0.21	3.2	234	0.002
Filled (F)	0.14 ± 0.72	0	2.1	234	0.037
Decayed (d)	0.08 ± 0.38	0	2.35	234	0.019
Filled (f)	0	0	0	0	—

**Table 2:** Comparison of DDQ between children with intellectual disability and normal children.

Question	Frequency	Disabled children		Normal children		Chi-square	P value
		n	%	n	%		
Problems with brushing upper teeth	Never	29	26.1	110	88	94.25	0.000^*^
	Occasionally	79	71.2	13	10.4		
	Always	3	2.7	2	1.6		

Problems with brushing lower teeth	Never	39	35.1	115	92	84.4	0.000^*^
	Occasionally	70	63.1	9	7.2		
	Always	2	1.8	1	0.8		

Puts away something nice to eat	Never	106	95.5	109	87.2	5.49	0.06^†^
	Occasionally	5	4.5	14	11.2		
	Always	0	0	2	1.6		

Bites with molar instead of front teeth	Never	100	90.1	95	76	9.27	0.010^*^
	Occasionally	10	9	22	17.6		
	Always	1	0.9	8	6.4		

Chewing at one side	Never	96	86.5	105	84	1.511	0.47^†^
	Occasionally	14	12.6	16	12.8		
	Always	1	0.9	4	3.2		

Problems in chewing	Never	95	85.6	115	92	7.62	0.022^*^
	Occasionally	16	14.4	7	5.6		
	Always	0	0	3	2.4		

Reaching for cheek while eating	Never	100	90.1	115	92	7.49	0.02^*^
	Occasionally	11	9.9	5	4		
	Always	0	0	5	4		

Crying at night	Never	109	98.2	119	95.2	2.28	0.31^†^
	Occasionally	2	1.8	4	3.2		
	Always	0	0	2	1.6		

Crying during meals	Never	102	91.9	121	96.8	2.72	0.09^†^
	Occasionally	9	8.1	4	3.2		
	Always	0	0	0	0		

Earache at night	Never	109	98.2	111	88.8	8.55	0.014^*^
	Occasionally	2	1.8	10	8		
	Always	0	0	4	3.2		

Earache at daytime	Never	110	99.1	114	91.2	7.76	0.02^*^
	Occasionally	1	0.9	7	5.6		
	Always	0	0	4	3.2		

Earache during eating	Never	111	100	115	92	9.27	0.01^*^
	Occasionally	0	0	8	6.4		
	Always	0	0	2	1.6		

Excessive salivation	Never	15	13.5	95	76	95.3	0.000^*^
	Occasionally	73	65.8	17	13.6		
	Always	23	20.7	13	10.4		

Putting hand inside the mouth	Never	45	40.5	116	92.8	74.24	0.000^*^
	Occasionally	63	56.8	9	7.2		
	Always	3	2.7	0	0		

Do you think your child has dental pain	Never	101	91	107	85.6		
	Always	9	8.1	18	14.4	3.35	0.18^†^
	Occasionally	1	0.9	0	0		

How frequent is your child's dental pain	Never	101	91	110	88	0.55	0.45^†^
	Occasionally	10	9	15	12		
	Always	0	0	0	0		

How severe is your child's dental pain	Never	102	91.9	66	52.8	45.21	0.000^*^
	Mild	6	5.4	50	40		
	Moderate	3	2.7	7	5.6		
	Severe	0	0	2	1.6		

When does pain occur	Never	102	91.9	66	52.8	45.33	0.000^*^
	Mild	5	4.5	48	38.4		
	Moderate	3	2.7	7	5.6		
	Severe	1	0.9	4	3.2		

^*^Statistically significant (P < 0.05).

^†^Statistically not Significant (P > 0.05).
